# The Impacts of Parental Migration on the Mental and Physical Health, Daily Needs, and Social Lives of Indonesian Caregivers of Left-Behind Children: A Qualitative Study

**DOI:** 10.3390/ijerph22081307

**Published:** 2025-08-20

**Authors:** Nelsensius Klau Fauk, Alfonsa Liquory Seran, Paul Russell Ward

**Affiliations:** 1 Centre for Public Health, Equity and Human Flourishing, Torrens University Australia, Adelaide 5000, Australia; 2 Atapupu Public Health Centre, Health Department of Belu District, Atambua 85752, Indonesia; liquory.seran@yahoo.com

**Keywords:** physical, mental and social well-being, basic and healthcare needs, financial condition, caregivers, left-behind children, parental migration, Indonesia

## Abstract

Despite its positive impact on household income, parental labour migration negatively affects left-behind children (LBC) and their caregivers. Studies in various settings have reported different impacts on LBC, but less is known about the consequences faced by their caregivers. This qualitative study used in-depth interviews to collect data from caregivers of LBC whose parents migrated for employment. Participants were recruited using the snowball sampling technique, starting with the distribution of study information sheets through village offices in Belu and Malacca districts, Indonesia. Data analysis was guided by a qualitative data analysis framework, which involved several steps, including familiarisation with the data or transcripts, identification of a thematic framework, indexing the data, charting the data, and mapping and interpreting the entire data. The findings showed that despite some benefits, including monthly remittances and positive feelings of living with and receiving support from LBC experienced by some caregivers, parental labour migration negatively impacted most caregivers due to their caregiving roles and responsibilities. These challenges included (i) mental and physical health issues, (ii) impacts on daily food and healthcare needs, and (iii) difficulties in their social lives and overall well-being. The findings underscore the need for comprehensive support systems and interventions to address these challenges and improve caregiver well-being. Such support systems should include access to mental and physical health services, financial assistance, employment opportunities, and social support networks. Future large-scale studies are recommended to explore the various impacts of parental migration and caregiving roles and responsibilities on caregivers of LBC, as the findings can better inform the development of policies and interventions to support them.

## 1. Introduction

Migration is a significant and complex global phenomenon, reflecting people’s efforts to seek safety or better employment and economic opportunities across borders. The 2021 report by the International Organisation for Migration estimated that there are 281 million international migrants worldwide, accounting for approximately 3.6% of the world’s population [1]. A significant portion of this number consists of labour migrants, who make up 60% or 169 million individuals, primarily driven by the pursuit of better employment opportunities abroad to improve their economic conditions [1,2]. As a result, labour migrants often come from low- and middle-income countries with limited employment opportunities.

Although the exact number of migrant workers who are parents is not available, parental migration to other places or countries for work has become a significant part of international labour migration. Consequently, millions of children around the world have been left behind, growing up without adequate care and support from their parents due to prolonged separation and reliance on family caregivers, such as grandparents, aunties, and uncles [3,4,5,6]. Studies in various settings globally have shown that these left-behind children (LBC) face numerous physical, psychological, and social challenges, making it difficult for them to navigate life without their parents [7,8,9,10]. This situation also imposes considerable difficulties and challenges on their caregivers, who must manage the complexities of raising children, deal with their emotional distress, and address their physical and social issues while also coping with their own burdens. This can lead to a caregiving burden that negatively impacts their emotional, physical, financial, and social functioning, given the high level of caregiving duties and the dependency of the children on them.

Limited evidence from some studies has reported that caregivers of LBC experience common mental disorders, such as depression, somatoform disorder, stress, and anxiety [5,6,11,12,13]. Some studies show that the high intensity of caregiving roles and responsibilities leads to caregiver stress [5,6,12], while others suggest that caregivers, particularly grandparents of LBC, face depression, somatoform disorders, and anxiety [11,13]. These studies identify various risk factors contributing to the mental health challenges faced by caregivers, including older age [5], which may diminish their ability to care for the children, and a lack of social support [12], leaving them feeling isolated in their caregiving roles. Infrequent contact from migrant parents and prolonged parental migration also contribute to these mental health challenges [11,13]. Additionally, low income among caregivers and irregular or absent remittances from migrant parents are significant risk factors for these mental health issues [5,11,13]. Caregivers with low income or poor economic conditions, along with limited resources, may face financial constraints that hinder their ability to meet the basic needs of their families, exacerbating their burden. Furthermore, some studies have reported that high-intensity caregiving negatively impacts caregivers’ physical health and accelerates the decline in their well-being, particularly for grandparent caregivers in rural areas [6,14]. For some elderly grandparent caregivers, the demands of caregiving are further intensified by challenges in managing their grandchildren’s rebellious attitudes and behaviours, adversely affecting their physical and mental health [6]. The rebellious attitudes and behaviours of LBC may indicate that they are experiencing mental stress and require additional emotional support in the absence of parental care.

Research on parental labour migration and its impact on LBC has expanded over the years in many settings [11,15,16,17]. However, a significant gap remains globally, including in the context of Indonesia, which is a limited understanding of the consequences of parental migration on caregivers who take on the new role and responsibilities of caring, raising, and nurturing LBC or how the caregiving role and responsibilities impact them. The existing literature highlights this critical gap, with several recent studies underscoring the scarcity of research focused specifically on caregivers of LBC with migrating parents [5,6]. This under-researched area calls for urgent scholarly attention, as understanding the dynamics and challenges faced by caregivers is essential for developing effective support systems and interventions. Therefore, this study aimed to fill this knowledge gap by exploring the multifaceted consequences of parental migration on Indonesian caregivers of LBC, focusing on their mental and physical health, daily necessities, health needs, and social life and well-being. This exploration was guided by the main research question: What are the impacts of parental migration on the caregivers of LBC? Understanding the impacts of parental labour migration on Indonesian caregivers of LBC is crucial for several reasons. Firstly, Indonesia is a major contributor to the international migrant workforce, with over four million Indonesian labour migrants employed in more than 25 countries worldwide [18]. The annual number of Indonesian migrant workers departing for overseas employment has increased in recent years, from 72,624 in 2020 to 274,964 in 2023 [19]. Secondly, there is no specific public social support available to support the needs of these caregivers and LBC. While exact figures for Indonesian migrant workers from Belu and Malacca districts are unavailable, existing information suggests they are among the highest contributors of labour migrants from the province [18,20,21]. The Central Bureau of Statistics reports that around 1000 to 2000 officially registered labour migrants from East Nusa Tenggara (ENT) province depart for overseas each year, including from Belu and Malacca [22]. This figure excludes thousands of illegal labour migrants from the province [23,24]. It is important to study these caregivers, as they are the primary caretakers and face the complex challenges of caring for and raising LBC. Understanding these challenges is crucial for developing supportive and comprehensive policies and interventions that address their unique needs and enhance the well-being of both the caregivers and LBC in various contexts.

## 2. Methods

The present study used the Consolidated Criteria for Reporting Qualitative Research (COREQ) framework to inform the methodology section of the report [25]. The COREQ checklist comprises 32 essential items designed to ensure explicit and comprehensive reporting of qualitative research, particularly interviews and focus groups.

### 2.1. Study Settings

This research was conducted in the districts of Belu and Malacca, ENT province, Indonesia. Malacca was part of Belu until its administrative separation in 2012. Both districts are relatively similar in population and geographical coverage. Belu covers an area of 1284.94 km^2^ and has a total population of 204,541, while Malacca covers 1160.63 km^2^ with a population of 171,079 [26,27]. Both districts have comparable healthcare infrastructure; Belu has three hospitals and 17 public health centres, while Malacca has one hospital and 17 community health centres [26,27]. Regarding labour migration, the 2022 data from the Indonesian Bureau of Statistics reported 57,167 migrant workers (including parents) from ENT, including Belu and Malacca districts, working abroad [18]. This study explores the impacts of parental labour migration on caregivers of LBC, which is less known in the context of Indonesia and globally. Belu and Malacca districts were selected for this research based on practical considerations, such as feasibility and familiarity with the areas.

### 2.2. Study Design, Participant Recruitment, and Data Collection

A qualitative design employing in-depth interviews was used to explore the perspectives and experiences of caregivers in Belu and Malacca districts, Indonesia, on how their engagement in the caregiving role and responsibilities following the migration of the parents of LBC affects them. This approach was used to gain in-depth and rich insights into the consequences on these caregivers’ mental and physical health, daily needs, health needs, and social life and well-being [28]. Participants were caregivers of LBC, whose parents had migrated for work in other places or countries. They were recruited using the snowball sampling technique because we did not have any lists of caregivers of LBC, which meant purposive or random sampling could not be undertaken. Instead, we relied on obtaining some initial participants for interviews through our professional networks in the study settings (village offices), and then we used the snowballing technique to expand our sample. Initially, the field researcher approached some village chiefs in the study settings to have an initial conversation about the possibility of distributing the study information sheets through their offices and recommending some initial potential participants. After receiving their permission, participant recruitment started by posting information sheets on information boards at their village offices. The field office staff also helped distribute the information to some potential participants and recommended that they participate. The study information sheets outlined this study’s aim, participant roles, data handling, and ethical approvals. Information on these aspects was provided to help the potential participants make their informed decision on whether to participate. Village offices were chosen as starting points for advertising the study information sheets because they often function as community centers in rural areas; thus, it was expected that the information about this study could be seen by community members. Potential participants who contacted and confirmed their participation were recruited and scheduled for interviews with the field researcher (ALS). This was followed by the snowball sampling technique, where initial participants were asked at the end of each interview to distribute the study information sheets to their eligible friends and families. Inclusion criteria included being: (i) a caregiver of one or more LBC, (ii) aged 18 or above, and (iii) willing to participate in this study voluntarily. A total of 22 caregivers (9 from Belu and 13 from Malacca) of LBC whose parents migrated for work were recruited and participated in the interviews. None of the participants who confirmed to participate withdrew from this study, and no repeat interviews were conducted with any of the participants.

Data were collected through face-to-face, in-depth interviews primarily in participants’ homes, with some held at village offices. Only the participant and the field researcher (ALS) were present in the interview room. The field researcher received formal training in qualitative research methods during her undergraduate study (S.Farm), has experience in public health research, including HIV-related topics and worked as a pharmacist at a public health centre in Belu. The interview venue and time were mutually agreed upon by each participant and the field researcher. Before the interviews, participants received verbal explanations about this study to ensure their understanding. The in-depth interviews were guided by a broad research question regarding the impacts of parental migration on the caregivers of LBC. This was then elaborated to explore several key areas such as caregivers’ experiences with caregiving roles and responsibilities for LBC, the impact of caregiving on their mental health, how caregiving tasks or daily activities affect their physical health, their experiences with basic needs and health needs, and how engagement in caregiving roles and responsibilities influences their social lives and well-being (see Supplementary Material S1: Interview guide). The interviews lasted approximately 45 to 60 min, were conducted in Indonesian and were audio-recorded, with field notes taken. All participants provided voluntary consent by signing and returning an informed consent form on the day of the interview. Participant recruitment and interviews continued until data saturation was achieved. Data saturation was determined by the researchers when responses from later participants mirrored earlier ones or did not yield new information. There were no established relationships between the researchers and any participants before this study. At the end of the interviews, each participant was offered the opportunity to comment on or correct their transcript after transcription by the researchers, but none requested to do so.

### 2.3. Data Analysis

The data analysis began by transcribing recorded interviews verbatim into coding sheets (NKF). Field notes were then integrated into the transcripts. Data were analysed manually in Indonesian to maintain the social and cultural context, with selected quotes translated into English for this publication [29]. The translation was performed by NKF and ALS, both fluent in Indonesian and English, and then reviewed by the senior author (PRW). Throughout the manuscript drafting process, the original Indonesian transcripts were repeatedly checked against the English translations to ensure accuracy [30]. Data analysis was guided by the steps of qualitative data analysis introduced by Ritchie and Spencer [31]. Firstly, the first author (NKF) read each transcript to understand its content and provide comments or labels on small sections (data extracts). The second author (ALS) then reviewed these comments and labels. Both authors discussed and refined the comments and labels during this process, which is referred to as *familiarisation* with the data. This was followed by the *identification of a thematic framework*, through which both authors started to identify ideas, issues, concepts, and themes from each transcript and list them. For example, ideas, concepts, or issues like “family obligation to take care of LBC”, “physical health impact”, “mental health challenges”, “family needs”, “health needs”, “educational needs of the children”, “social disengagement”, and “social well-being”, were listed and used to create a thematic framework. This thematic framework was then used to shift and sort the codes assigned to the data extracts. Next, each transcript was coded (*data indexing*) by NKF and then reviewed by ALS. It started with open coding of data extracts, which resulted in a long list of codes. This was followed by close coding to group similar codes under identified themes. For example, codes regarding responsibilities as part of the family to take care of the LBC, the matrilineal system, common practice of caring for children, positive feelings of living with and support by LBC, and remittance, were grouped under the theme: “Context and reasons why caregivers looked after the LBC”. Codes related to routine caregiving tasks and responsibilities, including cooking, cleaning, washing clothes, and accompanying children in learning and social activities, and how these factors affected caregivers physically and mentally, were categorised under the theme: “Mental and physical health challenges in the caregiving role and responsibilities for LBC”. The next step was *charting the data* by reorganising and rearranging the themes and their codes in a chart to enable comparison within each transcript and between transcripts. The final step was *mapping and interpretation,* where the data were mapped and interpreted as a whole [31]. It should be noted that these steps were often carried out simultaneously during the data analysis process, as the activities informed each other within and between the steps. Therefore, changes and refinements of codes and themes occurred throughout the data analysis process. The analysis incorporated both deductive reasoning, with themes derived from prior knowledge, and inductive reasoning, with themes emerging purely from the data. All authors were involved in regular discussions and revisions during data analysis and the manuscript writing process, and agreed on the final interpretation of the data as presented in this manuscript.

### 2.4. Ethical Consideration

This study received ethical approvals from Torrens University Australia and Krida Wacana Christian University in Indonesia. Before data collection, participants were informed about the study objectives, data confidentiality, and their right to withdraw without consequences through study information sheets provided during recruitment. The field researcher also provided verbal information to participants before the interviews to ensure their understanding and voluntary participation. Personal identifiers were removed from the transcripts to maintain anonymity. Participants were also offered free access to a counsellor arranged by the research team in case they experienced emotional discomfort during the interviews.

## 3. Results

### 3.1. Sociodemographic Profile of the Participants

A total of 22 caregivers of LBC in this study were aged between 47 and 74 years, with the majority in the 50–59 age group. All participants were farmers, with no paid jobs, primarily relying on agriculture and fishing to support their families’ daily needs. The majority had no formal education (*n* = 14), while the rest graduated from primary school (*n* = 7) and high school (see Table 1). Participants (and their partners) were the main caregivers for LBC for varying durations without substitutes. Half of the participants had been in the caregiving role for 3 months to 5 years, while the other half were in the role for 6 to 12 years since the children were left by their migrating parents. The 22 caregivers in this study took care of 36 LBC. The majority of the children (*n* = 28) were between 2 months and 5 years old when left by their parents, while the rest were between 6 and 9 years old.

### 3.2. Context and Reasons Why Caregivers Looked After the LBC

For all participants in this study, the roles and responsibilities for caring for LBC were not arranged through a formal procedure with the migrating parents. Similarly, there seemed to be no official agreement regarding the caregiving duration. For these participants, caring for the LBC, who were their grandchildren and nieces or nephews, was more of a family matter and was performed voluntarily, with some caregivers viewing it as part of their responsibility as a family. This was evident in statements like, “*If we don’t take care of her (the grandchild), who will*” (Caregiver 14), “*They are our grandchildren; they belong to us*” (Caregiver 20), and “*Even though they (LBC) are my sister’s children, they are just like my own children*” (Caregiver 9). The caregivers in this study were from the maternal side of LBC, who were the grandparents and aunties of the children. This reflects the matrilineal culture in the study settings, where children are part of the mother’s clan. Additionally, it is a common practice in the study settings for young children to live with and be cared for by their maternal grandparents or siblings (mostly sisters) in cases of maternal death or parental divorce, especially when the father remarries.

The caregivers’ willingness to voluntarily take on the caregiving roles and responsibilities for the LBC was also reflected in their approval for the parents’ decision to migrate for work. The underlying reasons for this approval included the challenging circumstances faced by the migrating parents at home (e.g., unemployment and some women divorced by their husbands) and the expectation that they would find jobs in the host areas or countries and earn an income for a better future for themselves and their families:

“*…here she just stayed at home, no work. Her husband divorced her and married another woman. … At that time, she knew some women who were about to leave to be labour migrants, and she wanted to go too. She told me, and I said OK because I thought it was better for her to go and find a job over there and earn income*”.

(Caregiver 3).

“*They (migrating parents) didn’t have jobs here. … So let them go and find jobs to improve their lives. It is fine that I take care of their child so that they can look for jobs elsewhere*”

(Caregiver 5).

For some caregivers, there were positive stories related to parental migration, reflected in the positive feelings of living with and receiving support from their children in their daily lives. Some stated, “*We like it that our grandchildren are with us every day. They are entertainment for us*” (Caregiver 19), and “*They grow up fast, now they also help us with household chores, such as cooking, washing dishes, …*” (Caregiver 13). Another positive aspect was reflected in the monthly remittance from some migrating parents (mentioned by four participants), as one participant said, “*They send us money every month to buy rice and other things and also for his (one of the kids) school needs*” (Caregiver 2). However, the overarching narratives from most caregivers show that the caregiving role and responsibilities had significant negative consequences on their mental and physical health, daily needs, healthcare needs, and social life and well-being. These findings were grouped into three main themes: (i) Mental and physical health challenges in the caregiving role and responsibilities for LBC, (ii) The impact on daily food and healthcare needs of caregivers’ families, and (iii) The challenges to social life and well-being of caregivers of LBC. A detailed explanation of each theme is provided below.

### 3.3. Mental and Physical Health Challenges in the Caregiving Role and Responsibilities for LBC

Parental labour migration often has consequences on other family members, such as grandparents, aunties, and uncles, who may have to take on caregiving roles and responsibilities for LBC. These caregiving roles and responsibilities can adversely affect their mental and physical health. Caregivers in this study reported significant burdens related to their routine tasks and responsibilities, contributing to various mental health challenges. For example, fully assuming parental role in caregiving, nurturing, and raising children for some years imposed substantial emotional demands, often leading to stress and worry. Similarly, concerns about daily life situations and the children’s well-being often caused stress and worry among them. The struggles and difficulties caregivers faced within their families, and seeing the children endure these hardships, also evoked feelings of stress, frustration, and burden. Therefore, for some, the ongoing demands of caregiving resulted in emotional exhaustion, as illustrated by the accounts of two caregivers who had been in the caregiving role for three and four years:

“*Since the time both of their parents left (became migrant workers), we (the woman and her husband) started to become parents all over again. We completely take over the duties and responsibilities of parenting, from waking up in the morning until late at night. They are our grandchildren, they are ours. But sometimes, there is a sense of stress, burden, and frustration, not only because of taking care of them but also because of the difficulties within our family. The children deserve to be happy and not have to experience these hardships*”

(Caregiver 4).

“*There is a feeling of worry and frustration seeing the condition of the children, who are still very young, and both of their parents have migrated, leaving them behind. They are very young and need full attention. When their parents first left to become migrant workers four years ago, it was very hard for us and the children as well. My wife and I felt frustrated and stressed because the burden was heavy. We could not sleep well at night, as the children often cried looking for their parents*”

(Caregiver 12).

The caregiving roles and responsibilities for children left behind by migrant parents are often immense, encompassing looking after and ensuring the children’s daily needs. In this study, these factors negatively affected the physical health of caregivers. The tasks were often intensive, involving daily routines such as cooking, cleaning, washing clothes, and even accompanying children in learning and social activities. The constant physical activities, without adequate time or opportunity for rest and recovery, led to physical exhaustion, which negatively affected caregivers’ health and overall quality of life. Additionally, the physical strain and declining health exacerbated mental health challenges, such as feelings of stress, frustration, and hopelessness. The following quotes from two caregivers, each caring for two LBC for several years, highlight these experiences and consequences:

“*I often feel exhausted, physically. Now, I am not feeling strong anymore. When his parents left, X (name of the child) was only 3 years old. I carried him morning, noon, and night for several years. In the first and second years, I didn’t get enough rest because X was so fussy. After two years, I started to feel physically weak and exhausted. …*”

(Caregiver 1).

“*The task of taking care of these children is quite intensive from morning to night and very physically tiring: cooking the food, feeding them, bathing them, watching them play, preparing them for school. Maybe because of physical exhaustion, I often feel emotional and angry at home… I also feel sorry for the children because they are still small. It seems like they remember their parents. Sometimes, they are gloomy and cry*”

(Caregiver 9).

The emotional and physical burdens were much heavier for caregivers over 60 years old caring for two or more children, especially those aged 1–5 years. This was due to the heightened attention required for young children, as well as the increased complexity and energy needed for daily activities. Moreover, advancing age further contributed to the caregiving burden as tasks and responsibilities became more demanding. These increased responsibilities often left caregivers physically exhausted from endless work and emotionally drained from the constant need to be available for the children:

“*I am now already 70 years old. In two months, I will be 71. I feel physically weaker and unable to take care of my two grandchildren. Sometimes I feel sorry thinking that if suddenly my husband and I get sick, then who will take care of them because their parents are not here*”

(Caregiver 17).

“*It feels quite heavy to take care of three grandchildren. I am 74 years old, so I often feel tired. It has been challenging because the two children are still little and need extra attention. Now, when the three of them get together, they often fight. Sometimes I am frustrated too … I have to supervise them from morning till night, non-stop*”

(Caregiver 15).

### 3.4. The Impact on Daily Food and Health Needs of the Caregivers’ Families

The roles and responsibilities of caring for the children also increased the needs of caregivers’ families, who had limited resources. Most caregivers struggled to meet their families’ daily food needs. They also faced challenges in fulfilling the educational needs of these children, such as paying for school fees, uniforms, and learning materials, which resulted in educational delays or school dropouts. These issues stemmed from the financial hardships caregivers faced due to factors like lack of permanent employment and income. Many caregivers, especially the elderly, relied on financial assistance or remittance from migrating parents, which was often inconsistent (the majority) or not at all. The uncertainty made it difficult for some caregivers to meet their daily food and children’s educational needs. Additionally, the rising cost of food without a stable income further exacerbated their situation:

“*We often have difficulty meeting our daily needs (food) because we do not have money. The rice harvest is also uncertain every year; sometimes, it fails. … In terms of money, we only rely on the money from them (migrating parents). They send money once every 4 or 5 months. The amount they send is only a little, so it is often not enough for food and drink every day, as well as the school needs of the little one and his snacks at school*”

(Caregiver 3).

“*We are farmers. It is very difficult to earn money in this village, which is why many people have migrated to find work elsewhere. … They (migrating parents) did not send money to support his education, and we had difficulty paying for his school needs. Nowadays, children have to bring pocket money to school every day. Where can we get the money? That is why he stopped going to school… The problem is that we also have difficulty meeting our daily needs. The price of food is also increasing…*”

(Caregiver 6).

Furthermore, the inability to access healthcare services for caregivers and left-behind children was another negative consequence of parental labour migration. Financial hardships and struggles experienced by caregivers hindered them from accessing health services for themselves and their children when they fell ill. Medical costs, including doctors’ fees, medications, and transportation, were often considered expensive for caregivers without a steady income or reliable remittance from migrant parents. Consequently, many relied on traditional medicine or home remedies, which were sometimes ineffective. This situation increased the risk of health complications due to inadequate treatment. The lack of access to healthcare not only increased the risk of health complications but also had emotional consequences, leaving caregivers feeling anxious and helpless in preserving their family’s well-being:

“*... If we are sick, we do not go to the doctor because we do not have money. If you calculate the costs of the motorcycle taxi, the doctor, and the medicine, it is too expensive. We cannot afford medical costs. … we use traditional medicines*”

(Caregiver 21).

Narratives from most caregivers in this study also highlighted a challenging situation in the study settings, with very limited employment options and alternative work choices they could take to support their lives. As one participant stated, “*In this village, it is difficult to find a job. Farming is the only option for me*” (Participant 2). Therefore, the additional caregiving roles and responsibilities for the children seemed to aggravate their already poor family conditions:

“*It is already difficult for us to meet our own needs every day. Now, you can imagine that we (the woman and her husband) also have to meet the needs of our two grandchildren. Hopefully, their parents send money. They have been gone for over a year, but so far, they have not sent any money*”

(Caregiver 18).

### 3.5. Challenges to the Social Life and Well-Being of Caregivers of Left-Behind Children

Caring for LBC also has consequences on the social lives of caregivers. Due to the demanding routine tasks of caring for the children, some participants lacked time to socialise with friends or engage in other social activities. Daily tasks, such as ensuring the children ate, studied, and rested properly, often left them little opportunity or time for activities outside the home. This situation led some caregivers to feel socially isolated, as they spent more time at home and rarely interacted with others. The lack of time for social or community events reduced their chances of receiving emotional support from others, further affecting their social well-being and sometimes left them feeling stressed and exhausted. Therefore, the disrupted social life increased the burden on caregivers, as illustrated by a grandmother caring for her grandchildren left by migrating parents:

“*I feel like I can’t go anywhere since they (migrating parents) left (migrated) because I have to take care of the children (LBC). They are still young, and I can’t leave them alone at home. In the past, I used to participate in a women’s group. We went on pilgrimages to the Maria cave, prayed together, went to the city and market, and participated in social activities at the village office. If there were social activities at the village or sub-district level, I always participated. Since A and B (names of LBC) started living with me, I have never participated. I cannot even attend wedding invitations. … I spend most of my time at home, sometimes I feel emotional and get angry …*”

(Caregiver 19).

Another participant, a grandfather, shared a similar experience, noting that his grandchild always followed him and insisted on going wherever he went. As a result, he had to give up a social activity he had enjoyed for years, which allowed him to meet and socialise with friends his age:

“*I used to participate in cockfighting twice a week, consistently. However, ever since they (migrating parents) migrated to Malaysia, I have stopped engaging in this activity, even though it was the only thing that brought me joy. This is an adult activity, so I cannot bring XXX (name of the child) along. He always cries and wants to go wherever I go*”

(Caregiver 22).

Furthermore, several participants expressed a lack of social support from friends, neighbours, community members, and even family, leaving them feeling overwhelmed by their roles and responsibilities. The absence of social support forced caregivers to manage their tasks alone, without assistance or a place to share their challenges. One of the reasons, as they recounted, is the possible societal perception that caring for children of migrating parents is a typical duty and responsibility within families, without necessitating support from others. This created unsupportive circumstances for caregivers, as portrayed in the following story:

“*It appears that people do not see the various burdens behind the duties and responsibilities of being a caregiver for the children. People perceive this as a normal part of family life. Perhaps because of this, we do not receive any support from others, friends, community members, neighbours, or even extended family members. No one asks about the difficulties or challenges we face in caring for children. People may think caring for these children is a common and problem-free task within families*”

(Caregiver 7).

The above narrative reflects the frustration of the participant due to a lack of understanding, sympathy, and support from their social environment. It also highlights a lack of understanding among community members about the complexity of the caregiving roles and responsibilities and the potential physical, emotional, and mental consequences for caregivers. This oversight results in caregivers being overlooked and receiving insufficient attention and support from others, including local governments.

## 4. Discussion

Parental labour migration, which is part of international migration, is a global phenomenon. However, current literature provides a limited understanding of the consequences for caregivers of children left by migrating parents. Our study highlights the significant challenges faced by caregivers of LBC, particularly regarding their mental and physical health, daily food needs, and social lives. The findings show that caregiving roles and responsibilities imposed substantial emotional demands on caregivers, leading to heightened emotional burden and exhaustion, including stress, worry, and frustration. These align with previous studies that report the associations between caregiving responsibilities and common mental health disorders, such as stress, depression, and anxiety, among caregivers [5,11,12,13]. What the current study adds to the existing knowledge is a more in-depth explanation of various risk factors, including pressure related to daily life situations with the children, concerns for their well-being, and difficulties within the household, all contributing to caregivers’ emotional exhaustion. These findings enrich those of previous studies, which have mainly reported associations between factors like older age [5], lack of social support [12], infrequent contact from migrating parents and prolonged parental migration [11,13], low income, and irregular or absent remittances [5,11,13] with mental health challenges faced by caregivers of LBC. Despite these findings, a previous study reported different results, suggesting that grandparent caregivers caring for grandchildren experienced fewer depressive symptoms than those who did not [32]. However, it is important to note that this study did not clarify whether the caregiving role improved caregivers’ mental well-being or whether those with better mental well-being were simply more effective in their caregiving responsibilities.

This study has also highlighted some findings on the physical health consequences of routine caregiving for LBC on caregivers, which have been minimally reported in previous studies [6,14]. Daily physical activities (e.g., cooking, cleaning, washing clothes, and accompanying children) without adequate time for rest and recovery led to physical exhaustion, negatively impacting caregivers’ physical health and exacerbating their mental health conditions, potentially diminishing their overall quality of life. These findings contribute to the limited understanding of how high-intensity care for LBC can result in physical health decline of caregivers [6,14]. Physical and mental health are integral to individuals’ well-being, hence, disruption in either aspect can negatively impact overall well-being [33]. Moreover, the current findings highlight that the caregiving burden was heavier for older caregivers caring for multiple LBC, especially those aged 1–5 years. This was due to the increased complexity and energy required for daily activities, coupled with advancing age, which further contributed to the caregiving burden in terms of both emotional and physical aspects. This aligns with a previous study’s finding suggesting an association between caregiving burden and stress among older caregivers [5]. The current findings have implications for policies and interventions to address the challenges faced by caregivers of LBC. There is a need for interventions focused on providing mental health support and resources for caregivers, especially older ones, such as counselling services and support groups, which are currently lacking in Indonesia and beyond. As the participants have been the main caregivers of LBC for extended periods without substitutes, it is also crucial for interventions to aim at reducing caregiving burdens by providing respite care or assistance with daily tasks. This could include programs offering temporary relief for caregivers, such as daycare centers or after-school programs, which currently do not exist in the study settings and other parts of Indonesia.

Our findings also suggest that caring for LBC has made it difficult for caregivers to meet daily necessities, such as food, clothing, educational needs of the children in their care, and family healthcare needs due to financial constraints. An increased number of family members and needs, reliance on inconsistent remittance, lack of resources, and limited income and job opportunities, which reflect a broader issue of limited employment in the study settings, were underlying reasons for the financial constraints faced by the caregivers. These financial hardships were further exacerbated by the rising cost of living, which stretched their already limited resources. Findings of a previous study have indicated that caregivers with higher annual incomes experienced less burden and stress associated with caregiving roles and responsibilities [5]. Thus, it is evident that higher family incomes can positively contribute to better living conditions and reduce the difficulties caregivers face in raising LBC. Our study also reveals that unaffordability and lack of access to healthcare not only exacerbated existing health issues of some caregivers but also increased their emotional burden, as they struggled to provide adequate care for themselves and the children in their care. These findings are consistent with previous studies suggesting that a lack of or limited access to healthcare services can lead to poor health outcomes among family members [34,35,36]. The current findings on the financial impact of parental migration are in contrast with previous findings reporting the positive impacts of parental migration on improving household financial conditions and supporting LBC’s education [37,38]. Our findings highlight the importance of addressing the economic challenges faced by caregivers and their families through interventions that provide access to stable employment and financial support. Therefore, the implications for the Indonesian government are clear; it needs to improve its existing policies on social support programs for families, such as non-cash food assistance, the Family Hope Program, direct cash assistance, food risk mitigation, and rice social assistance, to cover the families of caregivers of LBC [39,40]. Similarly, the findings call for the government of Indonesia to improve its health policy and healthcare services for caregivers and their families by expanding the coverage of the existing fully subsidised national health insurance [41]. Improvements at the policy level and in healthcare services are crucial, as the issue concerning the impact of migration on both LBC and their caregivers has not received adequate attention from either the Indonesian government or NGOs. This is reflected in the fact that there are currently no government policies, government aid, social support, or intervention programs specifically targeting the caregivers of LBC or the children themselves.

Our study reports a significant impact of caregiving roles and responsibilities on caregivers’ social lives, which has not been previously reported [11,12,13,42]. The overwhelming caregiving tasks often left caregivers with little time for socialising or participating in other social activities. This lack of social engagement not only intensifies the emotional burden, leading to feelings of stress and frustration, but also limits opportunities for relief from caregiving duties. Although only one previous study which has specifically reported an association between lack of social support and stress among grandparent caregivers of LBC [12], other studies have suggested that social disengagement and isolation can increase the risk of lower health status and mortality and are strongly correlated with depression in older adults [43,44]. Without a support system in place, caregivers seemed to be forced to manage their caregiving tasks and responsibilities alone for extended periods, resulting in both social and emotional burdens. The findings underline the importance of social networks, which have been shown to serve as sources of constructive exchanges and support that positively impact individuals’ health within the networks [45,46]. Therefore, interventions should focus on promoting social support networks and reducing social disengagement among caregivers of LBC. Community-based programs, such as support groups or social events, can provide opportunities for caregivers to connect and interact with others facing similar situations. Additionally, efforts should be made to raise awareness and understanding within families and communities about the challenges associated with caregiving to foster a more supportive and empathetic environment. Therefore, the role of the government and private entities, including NGOs that understand migration issues and their negative impacts on caregivers and LBC, is crucial and currently lacking.

### Limitations and Strengths of This Study

This study involved 22 caregivers of LBC in two rural districts of Belu and Malacca, Indonesia. Therefore, the findings reflect the specific perspectives and experiences of these caregivers, which may differ from those of caregivers in other settings with different characteristics. The authors realised that the snowball sampling technique used in this study and the small size of the villages where the participants were recruited might have introduced recruitment bias, as most participants in each village appeared to know each other. Therefore, the findings presented in this paper do not necessarily reflect the full picture of caregiving experiences in the study settings or beyond. Nevertheless, it is noteworthy that this study holds significance as it is the first qualitative research conducted in Indonesia that delves into the lived experiences of caregivers regarding the consequences on their mental and physical health, family and health needs, and social lives. Consequently, the insights from this research offer valuable guidance for developing policies and interventions aimed at addressing the challenges faced by caregivers and their families, as well as supporting their needs. It is imperative that future research continues to address these overlooked consequences of parental migration on caregivers of LBC to provide a more comprehensive understanding of the broader impacts of parental labour migration.

## 5. Conclusions

This study highlights the multiple challenges faced by caregivers of children left behind by migrating parents. Caregiving roles and responsibilities for these children were voluntary and seen as part of the family’s obligations. Routine tasks, such as cooking, cleaning, washing clothes, and assisting children with learning and social activities, contributed to physical exhaustion and mental health burdens. Engaging in caregiving responsibilities also increased family needs, causing struggles among caregivers to meet daily food requirements, cover children’s tuition fees, and address healthcare needs. The situation was exacerbated by their already precarious financial circumstances and a lack of employment opportunities. Routine caregiving tasks, coupled with insufficient social support, also adversely affected their social well-being, reflected in a lack of time for and withdrawal from social activities, which resulted in feelings of social isolation and frustration. This study also highlights the increased caregiving burden on older caregivers caring for multiple and younger LBC. Overall, the findings call for comprehensive support systems and interventions to address these challenges and improve caregivers’ well-being. These support systems should include access to mental and physical health services, financial assistance, employment opportunities, and social support networks. Future large-scale studies involving caregivers of LBC in different settings are recommended to investigate various impacts of parental migration and caregiving roles and responsibilities on them, as the findings can be compared to the current ones and better inform the development of policies and interventions to support them.

## Figures and Tables

**Table 1:** Sociodemographic profile of the caregiver participants.

No.	Sex	Age	Education	Relationship to LBC	Number of LBC Cared For	Duration of Being Caregivers (Year)
1	Female	50	No formal education	Grandmother	2	9
2	Male	56	Primary school	Grandfather	2	4
3	Female	47	Primary school	Grandmother	1	4
4	Female	65	No formal education	Grandmother	2	7
5	Female	47	Primary school	Grandmother	1	8
6	Female	56	No formal education	Aunty	1	5
7	Female	52	Primary school	Grandmother	1	6
8	Female	47	No formal education	Grandmother	1	<1
9	Female	52	No formal education	Aunty	2	2
10	Female	49	Primary school	Grandmother	1	3
11	Male	55	Primary school	Grandfather	2	8
12	Male	70	No formal education	Uncle	3	4
13	Female	59	No formal education	Grandmother	3	9
14	Female	56	Primary school	Grandmother	1	1
15	Female	74	No formal education	Grandmother	3	6
16	Female	54	No formal education	Grandmother	1	12
17	Female	70	No formal education	Grandmother	2	8
18	Female	53	No formal education	Grandmother	2	1
19	Female	60	No formal education	Grandmother	2	5
20	Male	54	Junior High School	Grandfather	3	8
21	Female	49	No formal education	Grandmother	1	6
22	Male	63	No formal education	Grandfather	1	4

## Data Availability

The data presented in this study are available on request from the corresponding author. The data are not publicly available due to restrictions set by the human research ethics committee.

## Supplementary Materials

The following supporting information can be downloaded at: https://www.mdpi.com/article/10.3390/ijerph22081307/s1, S1: Interview Guide.
