# Supplementary material for: The Impacts of Parental Migration on the Mental and Physical Health, Daily Needs, and Social Lives of Indonesian Caregivers of Left-Behind Children: A Qualitative Study
Source: Int J Environ Res Public Health. 2025 Aug 20;22(8):1307. doi: 10.3390/ijerph22081307 (PMC12386691; doi:10.3390/ijerph22081307)
Supplement: Supplementary file 1 [file ijerph-22-01307-s001.zip › ijerph-3715949-supplementary.pdf]

Interview Guide: Caregivers of Children Whose Parents Are Migrant Workers

Date: ______________________________
Interviewer: ____________________ (Name)

Initial Greeting…..

Thank you very much for agreeing to participate in this interview, and thank you for your time; I greatly appreciate it.

Is it alright if I record and take notes during the interview? When I write up the findings of this research, I will remove all identifying information about the informants to ensure that the information you provide in this interview remains anonymous. Is that acceptable to you? You can stop your participation or request to terminate the interview at any time if you do not wish to continue or feel uncomfortable during the interview, without any consequences.

As you have read in the information sheet and from our previous discussion when you confirmed your willingness to participate, the purpose of this research is to explore your experiences of caregiving roles and responsibilities and how these have impacted your life.

Demographic Information

Age :
Gender:
Level of Education : None/Primary/Secondary/High School/University
Who has migrated : Father or mother of the child, or both
Relationship to the child :

Employment :

Again, thank you for taking the time to meet with me today. I really appreciate your willingness to share your experiences.

- Could you please the me how many children are in your care at the moment?
    - Who are they?
    - How old are they?
- When did the parents of the children you care for migrate
    - To which place/country?

- How did you and the parents arrange for these caregiving roles and responsibilities you are taking on? Please explain.
    - Formal/informal agreement
    - Family matter/responsibility
- Could you please share your experiences of caregiving roles and responsibilities for the children? Please elaborate.
    - What roles and responsibilities
    - What routine tasks do you do in caring for the children? Please elaborate.
- How do you feel about the caregiving roles and responsibilities, as well as the routine tasks you perform in caring for the children? Please explain.
    - Do they affect you physically or mentally? How?
    - Your feelings about what the children have been going through – do they positively/negatively affect you? How?
- How do you manage to meet the needs of your family?
    - Daily needs for food
    - Healthcare needs
    - Children's education-related needs
- What kind of job are you engaged in?
    - Paid
    - Non-paid
- What is your source of income?
    - Is it sufficient?
    - Do you rely on remittance?
    - Please explain further
- Have you received any form of support from others to assist you in caring for the children? Please explain.
    - From neighbours
    - From friends
    - From non-governmental organisations
    - From the government

- Does engagement in caregiving roles and responsibilities influence your social lives and well-being? How? Please explain more.
    - No free time for social activities
    - Withdrawal from social activities
    - Social isolation
    - Etc….
